# Characterization of Clinical Isolates of *Talaromyces marneffei* and Related Species, California, USA

**DOI:** 10.3201/eid2509.190380

**Published:** 2019-09

**Authors:** Linlin Li, Katelyn Chen, Nirmala Dhungana, Yvonne Jang, Vishnu Chaturvedi, Ed Desmond

**Affiliations:** California Department of Public Health, Richmond, California, USA

**Keywords:** Fungi, Talaromyces marneffei, Penicillium marneffei, multigene phylogeny, MALDI-TOF, Talaromyces atroroseus, red pigment, California, opportunistic infections, United States, mass spectrometery

## Abstract

*Talaromyces marneffei* and other *Talaromyces* species can cause opportunistic invasive fungal infections. We characterized clinical *Talaromyces* isolates from patients in California, USA, a non–*Talaromyces*-endemic area, by a multiphasic approach, including multigene phylogeny, matrix-assisted laser desorption/ionization time-of-flight mass spectrometry, and phenotypic methods. We identified 10 potentially pathogenic *Talaromyces* isolates, 2 *T. marneffei*.

*Talaromyces marneffei* is a dimorphic fungal pathogen that causes focal or systemic infection in immunocompromised persons, primarily HIV-infected patients (*1*). Many cases have been reported in travelers returning from areas of Southeast Asia, southern China, and eastern India to which it is endemic. Other *Talaromyces* species also have been reported to cause invasive fungal infections, including *T. amestolkiae* (*2*), *T. purpurogenus* (*3*,*4*), and *T. piceus* (*5*,*6*). *Talaromyces* species are common in air, soil, and human habitats. Clinical laboratories in areas to which this fungus is not endemic often do not perform identification of *T*. *marneffei* and other *Talaromyces* species (*2*). Therefore, we devised a multiphasic approach for identifying *T. marneffei* and other potentially pathogenic *Talaromyces* species.

We conducted this study during 2018. *Talaromyces* isolates from 10 human specimens were submitted to the Microbial Diseases Laboratory (MDL), California Department of Public Health (Richmond, CA, USA), to rule out *T. marneffei* (Appendix). Temperature and pH are known to influence pigment production and colony morphology of *Talaromyces* species; therefore, growth characteristics were observed using 2 different culture media (Sabouraud dextrose agar, pH 5.6; and Sabouraud dextrose agar, Emmons, pH 6.9), incubated at 25°C and 30°C. Fungal DNA was extracted using a previously reported method (*7*). *Talaromyces* isolates were identified to species level using the internal transcribed spacer (ITS) region, partial β-tubulin gene (BenA), and partial RNA polymerase II largest subunit gene (RPB1) (*8*). The ITS and partial BenA and RPB1 sequences were used to search for homologies in GenBank and CBS databases (http://www.westerdijkinstitute.nl/collections). Multigene phylogenetic analysis was conducted on the concatenated ITS–BenA–RPB1 nucleotide sequence alignment (Appendix). A blastn search (https://blast.ncbi.nlm.nih.gov/blast) through the GenBank database, pairwise comparison alignment through the CBS database, or both showed 99%–100% homology for ITS, 97%–100% for BenA, and 91%–100% for RPB1 sequences with the best-matched sequences of known *Talaromyces* species isolates.

Phylogenetic analysis of the *Talaromyces* isolates showed 7 genetic clades, consistent with previous descriptions of the *Talaromyce*s genera (*9*) (Figure). Species identification using a comparison of the ITS, BenA, and RPB1 sequences with existing sequences and multigene phylogenetic analysis identified *T. marneffei* (isolates MDL17022 and MDL18026), *T. atroroseus* (MDL17026, MDL17144, MDL17164, and MDL18070), *T. islandicus* (MDL18167), *T. stollii* (MDL18054), *T. coalescens* (MDL18102); and *T. australis* (MDL18159). The 2 *T. marneffei* isolates produced diffuse red pigment early, by 3 days of growth, on both medium types and at both incubation temperatures. *T. australis* and *T. stollii* isolates also produced red pigment by 3 days but with variations based on media or temperature. At 7 days of growth, the 4 *T. atroroseus* isolates also showed variable red pigment production (abundant, weak, and absent) (Appendix). Microscopically, most isolates showed biverticillate conidiophores and globose to fusiform conidia in unbranched chains. Both *T. marneffei* isolates were from HIV-positive patients. MDL17022 was from a blood sample of a 37-year-old man with a travel history to Southeast Asia; MDL18026 was from skin tissue of a 36-year-old man with no available travel history.

**Figure:**
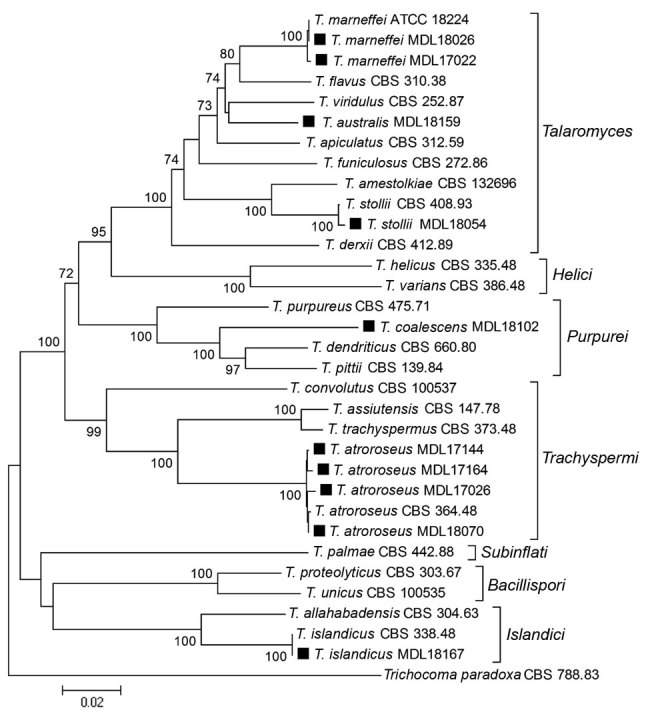
Phylogenetic analysis of *Talaromyces* species based on concatenated nucleotide alignments of internal transcribed spacer, partial β-tubulin gene, and partial RNA polymerase II largest subunit gene regions, showing the relationship among clinical isolates from patients in California, USA (black squares), and reference *Talaromyces* species. The tree was constructed by the neighbor-joining method with 1,000 bootstrap replicates by using MEGA software (https://www.megasoftware.net). Bootstrap support values >70% are presented at the nodes. The tree was rooted with *Trichocoma paradoxa* CBS 788.83. GenBank accession numbers for newly generated sequences are MK601832–41 for the internal transcribed spacer, MK626499–508 for the β-tubulin gene, and MK626509–518 for the RNA polymerase II largest subunit gene. CBS, Westerdijk Fungal Biodiversity Institute; MDL, Microbial Diseases Laboratory, California Department of Public Health. Scale bar indicates estimated phylogenetic divergence.

Using matrix-assisted laser desorption/ionizationtime-of-flight (MALDI-TOF) mass spectrometry, we generated main spectrum profiles (MSP) of *Talaromyces* species following Bruker’s custom MSP and library creation standard operating procedure (https://www.bruker.com). We extracted proteins of *Talaromyces* isolates using the previously published National Institutes of Health (NIH) protocol (*10*). We analyzed *Talaromyces* spectra with MALDI Biotyper 4.1 software against combined databases of the Filamentous Fungi Library 2.0 (Bruker) and the NIH Mold Library (*10*), with and without inclusion of newly created MSPs of *Talaromyces* species (Appendix). The threshold for species identification was >1.9; for genus identification, >1.7. 

Using the combined databases of Filamentous Fungi Library 2.0 (Bruker) and NIH Mold Library, we identified none of the isolates to species level; results showed either no identification or genus-level identification. However, when we expanded the combined database with the MDL Mold Library, we correctly identified all *Talaromyces* isolates to the species level with the best score >1.9. There were no ambiguous identification results; that is, the second-best matched species also had a high confidence score >1.9.

*T. marneffei* can be readily differentiated from other red pigment–producing *Talaromyces* species by yeast-like colony conversion at 37°C. However, many clinical laboratories no longer conduct yeast conversions. For those laboratories, yellow-green colonies producing red soluble pigment at ≈3 days on common fungal culture media at 25°C–30°C might indicate the need to further confirm *T. marneffei*. It is difficult to distinguish *Talaromyces* species only by macroscopic and microscopic examination. Multilocus sequencing, although confirmatory, might be too time-consuming and expensive for routine use. Therefore, we identified all *Talaromyces* isolates to species level by MALDI-TOF mass spectrometry by using an expanded database with well-characterized *Talaromyces* strains. 

In conclusion, our results show that MALDI-TOF mass spectrometry is a good choice for rapid, less expensive primary identification of *Talaromyces* species and other medically important fungal pathogens. Species-level identification of *Talaromyces* isolates is clinically useful for treatment of patients with underlying conditions, such as immunodeficiency, cancer, advanced age, and immunosuppressive therapy.

Appendix Additional details for clinical isolates of *Talaromyces marneffei* and related species, California, USA.
