# Supplementary material for: Characterization of Clinical Isolates of Talaromyces marneffei and Related Species, California, USA
Source: Emerg Infect Dis. 2019 Sep;25(9):1765–8. doi: 10.3201/eid2509.190380 (PMC6711226; doi:10.3201/eid2509.190380)
Supplement: Appendix — Additional details for clinical isolates of Talaromyces marneffei and related species, California, USA. [file 19-0380-Techapp-s1.pdf]

Article DOI: https://doi.org/10.3201/eid2509.190380

Clinical Isolates of *Talaromyces marneffei* and Related Species, California, USA

Appendix

Appendix Table 1. Source of *Talaromyces* isolates

Isolate ID	Culture source	Age/sex	Species
MDL17022	Blood sample	37/M	*T. marneffei*
MDL17026	Sputum sample	53 /F	*T. atroroseus*
MDL17144	Ear	9 /M	*T. atroroseus*
MDL17164	Nail	85/M	*T. atroroseus*
MDL18026	Skin tissue	36/M	*T. marneffei*
MDL18054	Sputum	52/F	*T. stollii*
MDL18070	Bronchial wash	73/F	*T. atroroseus*
MDL18102	Bronchoalveolar lavage sample	59/F	*T. coalescens*
MDL18159	Bronchoscopy sample	72/F	*T. australis*
MDL18167	Bronchial wash	63/M	*T. islandicus*

Appendix Table 2. Identification of *Talaromyces* isolates by matrix-assisted laser desorption/ionization–time of flight mass spectrometry*

		Bruker and NIH database†		Bruker, NIH, and MDL database	
Isolate	Species ID by sequencing	Best match	Best score	Best match	Best score
MDL17022	*T. marneffei*	*T. marneffei*	1.22	*T. marneffei*	2.31
MDL17026	*T. atroroseus*	No ID	–	*T. atroroseus*	2.18
MDL17144	*T. atroroseus*	No ID	–	*T. atroroseus*	2.30
MDL17164	*T. atroroseus*	No ID	–	*T. atroroseus*	2.57
MDL18026	*T. marneffei*	No ID	–	*T. marneffei*	2.17
MDL18054	*T. stollii*	*T. ruber*	1.78	*T. stollii*	2.36
MDL18070	*T. atroroseus*	No ID	–	*T. atroroseus*	2.23
MDL18102	*T. coalescens*	No ID	–	*T. coalescens*	2.02
MDL18159	*T. australis*	*Talaromyces spp.*	1.38	*T. australis*	2.31
MDL18167	*T. islandicus*	No ID	–	*T. islandicus*	2.02

*ID, identification; MDL, Microbial Diseases Laboratory, California Department of Public Health; NIH, National Institutes of Health. Dash indicates .
†Bruker Filamentous Fungi Library 2.0 and NIH Mold Library (Lau AF, Drake SK, Calhoun LB, Henderson CM, Zelazny AM. Development of a clinically comprehensive database and a simple procedure for identification of molds from solid media by matrix-assisted laser desorption ionization-time of flight mass spectrometry. J Clin Microbiol. 2013;51:828–34).

Appendix Table 3. Growth study of *Talaromyces* isolates*

					Red soluble pigment†							
					3 d				7 d			
		37°C growth	Colony characteristics on SDA 30°C		SDA		E-SDA		SDA		E-SDA	
Isolate ID	Species ID	on SDA	3 d	7 d	25	30	25	30	25	30	25	30
MDL17022	*T. marneffei*	+	Yellowish white, velvety	Yellowish green, reddish brown, floccose	+	+	+	+	+	+	+	+
MDL17026	*T. atroroseus*	+	Blue green, felty	Blue green, floccose, white center	–	–	–	–	–	–	–	–
MDL17144	*T. atroroseus*	+	White, downy	Dark blue green, velvety, red center	–	–	–	–	–	+	+	+
MDL17164	*T. atroroseus*	+	White, downy	Dark blue green, floccose, yellow center	–	–	–	–	–	+	–	–
MDL18026	*T. marneffei*	+	Yellowish green, powdery	Yellowish green, pinkish brown, floccose	+	+	+	+	+	+	+	+
MDL18054	*T. stollii*	+	White, floccose	White, floccose	–	–	+	–	+	–	+	–
MDL18070	*T. atroroseus*	+	White, downy	Blue green, reddish brown, floccose	–	–	–	–	+	+	+	–
MDL18102	*T. coalescens*	–	Whitish green, downy	Whitish-green, floccose	–	–	–	–	–	–	–	–

					Red soluble pigment†							
					3 d				7 d			
		37°C growth on SDA	Colony characteristics on SDA 30°C		SDA		E-SDA		SDA		E-SDA	
Isolate ID	Species ID		3 d	7 d	25	30	25	30	25	30	25	30
MDL18159	*T. australis*	+	White, red, floccose	Red, floccose	–	+	+	+	+	+	+	+
MDL18167	*T. islandicus*	+	Green, orange, felty	Dark green, felty, orange edge	–	–	–	–	–‡	–‡	–‡	–‡

*E-SDA, Sabouraud dextrose agar, Emmons; SDA, Sabouraud dextrose agar; +, positive ; –, negative.
†Red soluble pigment producing was observed at 25°C and 30°C.
‡*T. islandicus* produces yellow-orange soluble pigment.

Appendix Table 4. *Talaromyces* isolate reference sequences and their GenBank accession numbers*

Species	Collection no.	ITS	BenA	RPB1
T. allahabadensis	CBS 304.63	KF984873	KF984614	JN680309
T. amestolkiae	CBS 132696	JX315660	JX315623	JX315679
T. apiculatus	CBS 312.59	JN899375	KF741916	JN680293
T. assiutensis	CBS 147.78	JN899323	KJ865720	JN680275
T. atroroseus	CBS 364.48	KF114740	KF114790	KF114750
T. convolutus	CBS 100537	JN899330	KF114773	JN121553
T. dendriticus	CBS 660.80	JN899339	JX091391	JN121714
T. derxii	CBS 412.89	JN899327	JX494306	JN680306
T. flavus	CBS 310.38	JN899360	JX494302	JN121639
T. funiculosus	CBS 272.86	JN899377	JX091383	JN680288
T. helicus	CBS 335.48	JN899359	KJ865725	JN680300
T. islandicus	CBS 338.48	KF984885	KF984655	JN121648
T. marneffei	ATCC 18224	NW_002196683	NW_002196666	NW_002196662
T. palmae	CBS 442.88	JN899396	HQ156947	JN680308
T. pittii	CBS 139.84	JN899325	KJ865728	JN680274
T. proteolyticus	CBS 303.67	JN899387	KJ865729	JN680292
T. purpureus	CBS 475.71	JN899328	GU385739	JN121687
T. stollii	CBS 408.93	JX315674	JX315633	JX315693
T. trachyspermus	CBS 373.48	JN899354	KF114803	JN121664
T. unicus	CBS 100535	JN899336	KJ865735	JN680324
T. varians	CBS 386.48	JN899368	KJ865731	JN680305
T. viridulus	CBS 252.87	JN899314	JX091385	JN680284
Trichocoma paradoxa	CBS 788.83	JN899398	KF984556	JN121718

*ATCC, American Type Culture Collection; BenA: β-tubulin gene; CBS, Westerdijk Fungal Biodiversity Institute, Utrecht, the Netherlands; ITS, internal transcribed spacer; RPB1, RNA polymerase II largest subunit gene.
